# Comparison of Salt Stress Tolerance among Two Leaf and Six Grain Cultivars of *Amaranthus cruentus* L.

**DOI:** 10.3390/plants12183310

**Published:** 2023-09-19

**Authors:** Adrien Luyckx, Stanley Lutts, Muriel Quinet

**Affiliations:** Groupe de Recherche en Physiologie Végétale, Earth and Life Institute-Agronomy, Université Catholique de Louvain, 1348 Louvain-la-Neuve, Belgium; adrien.luyckx@uclouvain.be (A.L.); stanley.lutts@uclouvain.be (S.L.)

**Keywords:** abiotic stress, amaranth, orphan crop, plant physiology, pseudocereal, salinity

## Abstract

Amaranths (*Amaranthus* L.) are multi-use crop species renowned for their nutritional quality and their tolerance to biotic and abiotic stresses. Since the soil salinity of croplands is a growing problem worldwide, we tested the salinity tolerance of six grain and two leaf cultivars of *Amaranthus cruentus* L. The plants were grown for 53 days under hydroponic conditions at 0, 50 and 100 mM NaCl. We investigated the growth rate, photosynthetic activity, mineral content, pigments and biochemical compounds involved in oxidative stress. Although 100 mM NaCl always decreased biomass production, we highlighted Don Leon and K91 as tolerant cultivars under moderate salt stress (50 mM NaCl). Under salinity, sodium accumulated more in the shoots than in the roots, particularly in the stems. Sodium accumulation in the plants decreased the net photosynthetic rate, transpiration rate and stomatal conductance but increased water use efficiency, and it decreased chlorophyll, betalain and polyphenol content in the leaves. It also decreased the foliar content of calcium, magnesium and potassium but not the iron and zinc content. The physiological parameters responded differently to sodium accumulation depending on the cultivar, suggesting a different relative importance of ionic and osmotic phases of salt stress among cultivars. Our results allowed us to identify the morpho-physiological traits of the cultivars with different salt tolerance levels.

## 1. Introduction

In a context of the increasing food demand and more adverse biotic and abiotic conditions for agricultural production, it is necessary to rely on robust crops that are able to thrive in stressful conditions, while limiting agriculture’s contribution to global change [1]. Currently, soil salinity is one of the main abiotic stress threatening agricultural production worldwide [1].

Soil salinization, caused by an accumulation of soluble salts (mainly NaCl and Na_2_SO_4_) in the upper horizon of soil, is an expanding agronomic constraint for food production, especially in Asia, Africa, South America and Australasia in arid regions where precipitations are too low to leach excessive salts [2,3]. A soil is saline when its electrical conductivity is higher than 4 dS·m^−1^ and sodic when the exchangeable sodium percentage is higher than 6% [4]. Salinization can be primary when it is of natural origin (weathering of saline rocks, saline bedrock, atmospheric deposition) or secondary when it is human-induced. In the latter case, saline or sodic soils can be the result of bad irrigation practices (e.g., with brackish water), sea level rising or an excessive use or bad management of mineral fertilizers [5]. Saline and sodic soils are estimated to cover more than 800 Mha, but we are lacking recent data [6,7]. Most of the salt-contaminated topsoils (>400 Mha, 0–30 cm) are saline (85%), whereas a much lower proportion is sodic or saline–sodic (15%) [6]. Salt-affected subsoils are twice as common as salinized topsoils (>800 Mha) and a higher proportion of them are sodic or saline–sodic (38%) [6]. Salt-affected soils are in expansion in part because of climate change and this issue is particularly exacerbating under arid and semi-arid climates and on irrigated croplands [5].

Salt stress in plants occurs in two phases, namely the osmotic and the ionic phases [8]. First, the osmotic stress is driven by the low hydric potential of saline soils that decreases water uptake. Osmotic stress has a direct effect on plant growth. Then, the ionic stress is caused by the toxicity of salt ions entering the plant tissues and the competitive effect with important nutrients. Tolerance mechanisms consist of, depending on the species, salt exclusion or salt accumulation involving tissue partitioning and subcellular compartmentation, and the synthesis of organic osmolytes for osmotic adjustment [8,9,10]. These tolerance mechanisms consume energy, which impedes growth and development [11].

Most major crops, which provide most of the world’s calories, are salt-sensitive even though extensive research is in progress to increase their salt tolerance [12,13,14]. Therefore, the use of so-called “orphan” or “indigenous” crops and wild crop relatives (WCR) has been flourishing in the last few decades [15,16,17,18]. It is a large research field with different approaches, including the introgression of genes from orphan crops or WCR to major crops, breeding of under-domesticated crops or de novo domestication of wild species [19,20,21,22]. This array of approaches is promising for breeding for salinity tolerance [22,23]. The amaranth genus (*Amaranthus* spp.) contains several orphan crops and WCR that has gained increasing attention in the past few decades because of its tolerance to abiotic and biotic stress, including salinity.

*Amaranthus* is a subcosmopolitan genus of 50–70 herbaceous plant species, most of which are annual plants, in the Amaranthaceae family [24]. Several species of amaranths are used as crops, either as leafy vegetables for their nutritious leaves or as pseudocereals for their quinoa-like seeds rich in high-quality proteins. Although some species were staple crops in several Mesoamerican civilizations, they fell into disuse for several centuries after european colonization of the Americas. However, a surge in interest for amaranths has arisen in the last few decades because of their nutritive qualities [25,26,27] and their tolerance to several biotic and abiotic stresses. Among grain species, studies have been conducted on salinity [28,29,30,31,32,33,34] and drought [35,36] in *A. cruentus*, on salinity in *A. caudatus* [37,38] and salt and drought in *A. hypochondriacus* [39,40]. The plant response to abiotic stress has also been investigated in some leaf species, such as *A. tricolor* [41,42,43] or *A. hybridus* and *A. albus* [44]. Most of these studies show that amaranth is highly tolerant to drought and salinity, particularly at the vegetative stage. These works report variability among species and among cultivars of each species. It has been shown that gas exchange regulation, antioxidant defense, ion transporter regulation and osmotic adjustment are involved in the stress response. Amaranths express the NAD malic-type C_4_ photosynthetic pathway, which makes them more competitive in warm and/or dry environments by means of an higher water use efficiency compared to C_3_ plants [45]. *Amaranthus cruentus* L. (red amaranth), domesticated by the Aztecs in Mesoamerica, is used either for leaves (in Africa and South-East Asia but also in America [46]) or for seed production (mainly in America and Asia, but also in Africa [47]), usually with distinct cultivars [48,49,50]. However, this species, along with the two other grain amaranths (*A. hypochondriacus* L. and *A. caudatus* L.), are promising as dual-use crops, with leaves and seeds harvested on the same plants [51,52,53]. In these three ‘grain’ species, the nutritional quality of both leaves and seeds is high. Seeds are rich in proteins, more than most true cereals, and minerals [54,55], while leaves are also rich in proteins, minerals and vitamins [56,57,58]. Grain amaranth can produce seed yields of 2000–3500 kg·ha^−1^ [59], whereas leaf amaranth can produce several dozens of tons per hectare of fresh leaves and young stems eaten as vegetables [60]. Despite their tolerance to salinity and their exceptional agronomic and nutritional value, amaranths have remained understudied crops. Indeed, the physiological mechanisms underlying abiotic stress tolerance are poorly known.

To deepen our understanding of the salt response in *Amaranthus cruentus* at the vegetative stage, a screening of the salt tolerance of six grain and two leaf cultivars was conducted under hydroponic conditions at moderate and strong levels of salt stress (50 mM and 100 mM NaCl, respectively [61]). Plant growth and photosynthetic activity were monitored. Na and K were quantified in leaves, stem and roots. In addition, other mineral contents (Ca, Fe, Mg and Zn) were determined in leaves since Na accumulation in plants is known to affect mineral nutrition [41]. The pigments (chlorophylls, betalains) and biochemical compounds involved in oxidative stress (malondialdehyde, polyphenols, flavonoids and ascorbate) were investigated in the leaves due to their importance in stress response [62]. The aims were to identify (1) contrasted cultivars regarding their tolerance to salt stress and (2) the main physiological mechanisms explaining variability in salt tolerance among leaf and grain cultivars of *A. cruentus*.

## 2. Results

Plants were subjected to 0, 50 and 100 mM NaCl for 53 days. The eight cultivars differed by their origin, morphology, color and food purpose (Table S1). The plants tolerated the salt stress well. Indeed, the mortality rate was low. Complete senescence was observed at 100 mM NaCl only in two plants of Montana 5 and two plants of Don Leon, for a total of 10 plants per cultivar and condition.

### 2.1. Biomass Production

**Figure 1:**
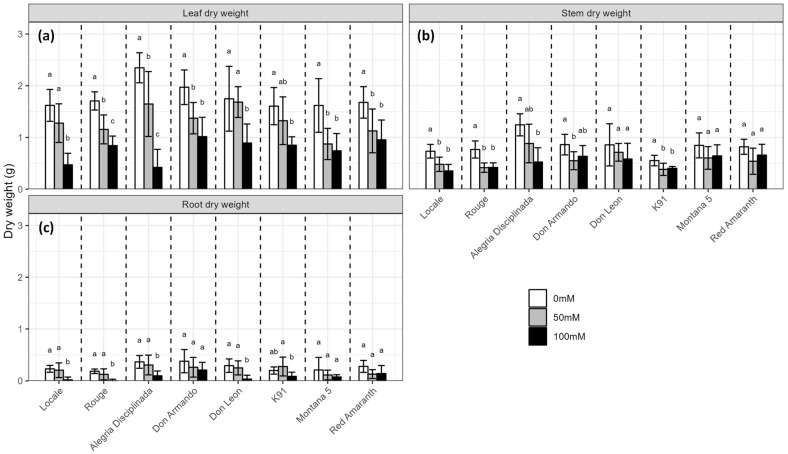
Effect of salinity (0, 50 and 100 mM NaCl) on the dry biomass production of eight *A. cruentus* cultivars after 53 days of growth. (**a**) Leaf dry weight; (**b**) stem dry weight; (**c**) root dry weight. Treatments followed by different letters for the same cultivar are significantly different (*p* < 0.05).

Under the control conditions, the mean leaf dry weight of all cultivars was 1.79 ± 0.43 g. The most productive cultivars were Alegria Disciplinada and Don Armando, whereas the less productive ones were K91, Montana 5 and Locale (Figure 1). Total dry weight was strongly correlated with leaf dry weight (r = 0.98), with leaves accounting for 62.5 ± 3.8% of the total dry biomass, whereas stem and roots accounted for 28.6 ± 4% and 8.8 ± 3.5%, respectively.

Salt decreased the dry weight of all organs (*p* < 0.001 for leaves, stem and roots) in all cultivars (Figure 1, Table S2). The leaf dry weight decreased by on average 57% at 100 mM NaCl (Figure 1a). The strongest effect was observed on Alegria Disciplinada (−82%), which was the most productive without salt. Red Amaranth and K91 were the least affected, with less than 50% of leaf weight loss. At 50 mM NaCl, salt caused an average decrease in leaf biomass of 27%, but with some variability among cultivars. Don Leon leaf dry weight was hardly affected by 50 mM NaCl, whereas Montana 5 leaf production dropped by 46%. Moreover, the leaf production of Don Armando, K91, Montana 5 and Red Amaranth was similar at 50 and 100 mM NaCl.

Stem dry weight decreased significantly at 50 mM NaCl only in Locale, Rouge, K91 and Don Armando, whereas it was not affected at 100 mM NaCl in Montana 5, Don Leon, Don Armando and Red Amaranth (Figure 1b). Root dry weight rarely decreased at 50 mM NaCl, but often strongly at 100 mM NaCl, by more than 70% in Alegria Disciplinada and even more than 80% in Don Leon (Figure 1c).

Salt stress slightly influenced the stem and root water content (*p* = 0.004 and *p* = 0.049, respectively), which were 95 ± 1% and 97 ± 3% in control conditions, respectively (Tables S2 and S3). The leaf water content was 88 ± 1% in control conditions. While no effect was observed at 50 mM NaCl, salt substantially decreased the leaf water content at 100 mM NaCl, which dropped to 81 ± 13%. This decrease was only significant in Montana 5 (−12.3%), Alegria Disciplinada (−16.2%) and Don Leon (−17.9%).

Based on the DW, the salt tolerance index (STI) was calculated (Table 1). At 50 mM NaCl, the most sensitive cultivar to salt was Montana 5, whereas Don Leon was the most tolerant. Cultivars behaved substantially differently at 100 mM NaCl, since Alegria Disciplinada was the most sensitive and Red Amaranth the most tolerant. Indeed, the tolerance index did not demonstrate any correlation at 50 mM and 100 mM NaCl (r = −0.11, *p* = 0.80).

### 2.2. Sodium Distribution in the Plant Organs

**Figure 2:**
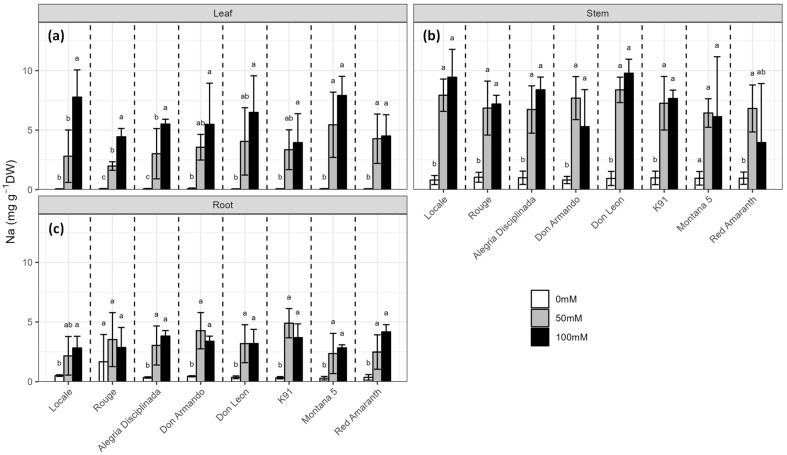
Effect of salinity (0, 50 and 100 mM NaCl) on the sodium content in (**a**) leaves; (**b**) stems and (**c**) roots of the eight *A. cruentus* cultivars. Treatments followed by different letters for the same cultivar are significantly different (*p* < 0.05).

Salt stress significantly increased Na concentrations in the leaves (*p* < 0.001), stems (*p* < 0.001) and roots (*p* < 0.001) but no differences were observed among different cultivars (Figure 2, Table S4). The sodium content in stems and roots was similar between 50 mM and 100 mM NaCl, whereas the accumulation was proportional to the stress intensity in leaves, especially in Locale, Rouge and Alegria Disciplinada. Salt stress caused an accumulation of Na in leaves up to 5.45 ± 2.74 mg·g^−1^ DW in Montana 5 at 50 mM NaCl and up to 7.91 ± 1.61 mg·g^−1^ DW at 100 mM NaCl in the same cultivar (Figure 2a). Rouge had the lowest accumulation of Na in leaves at 50 mM NaCl with 1.99 ± 0.36 mg·g^−1^ DW; at 100 mM NaCl, the lowest accumulation was observed in K91 (3.95 ± 2.43 mg·g^−1^ DW). Sodium accumulated in stems at a higher magnitude than in leaves (up to 8.38 ± 1.07 mg·g^−1^ DW at 50 mM NaCl and up to 9.78 ± 1.17 mg·g^−1^ DW at 100 mM NaCl, in both cases in Don Leon, Figure 2b). In contrast to stems, Na accumulated in roots at lower concentrations than in leaves (up to 4.90 ± 1.23 mg·g^−1^ DW in K91 at 50 mM NaCl and up to 4.17 ± 0.60 mg·g^−1^ DW in Red Amaranth at 100 mM NaCl, Figure 2c).

### 2.3. Overview of the Physiological Response of the Cultivars to Salt Stress

**Figure 3:**
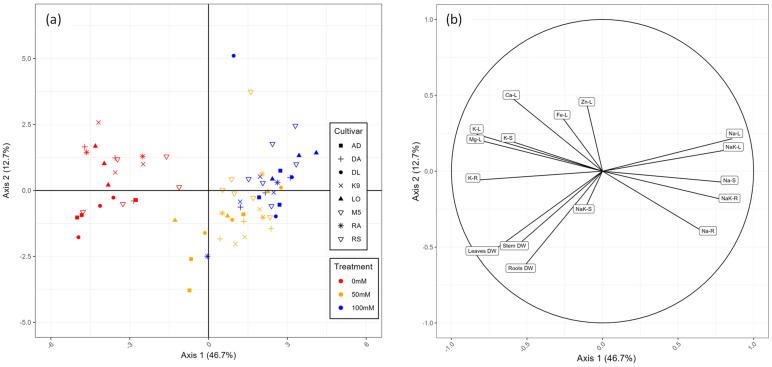
Principal component analysis (PCA) of the plant growth and mineral content of eight *A. cruentus* cultivars exposed to 0 mM, 50 mM and 100 mM NaCl. (**a**) Individual plot showing the eight cultivars position (LO, Locale; RO, Rouge; AD, Alegria Disciplinada; DA, Don Armando; DL, Don Leon; K9, K91; RA, Red Amaranth; M5, Montana 5) in the three salt treatments (red, 0 mM; yellow, 50 mM; blue, 100 mM). (**b**) Variable plot showing correlations between mineral content and biomass data (DW, dry weight; -L, leaf; -S, stem; -R, root).

**Figure 4:**
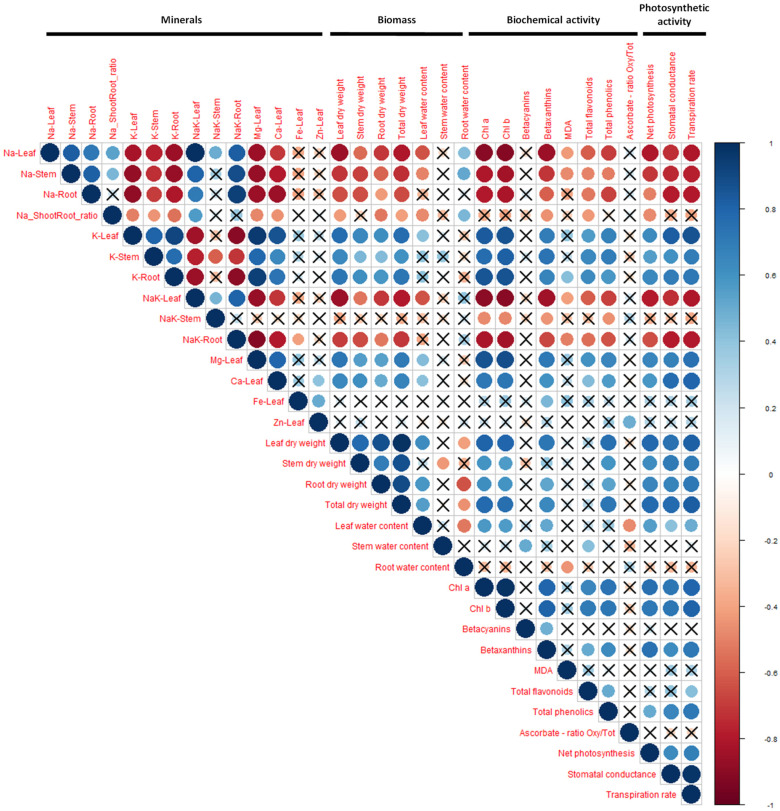
Correlation graph of all measured parameters, grouped in four categories: mineral content, biomass production, pigments, biochemical activity in leaves and photosynthetic activity. Non-significant (*p* < 0.05) correlations are crossed out. Negative correlations are colored in shades of red, whereas positive correlations are in blue (see the color legend on the right). Na_Shoot and Root_ratio, ratio between the quantity of sodium in the shoot to the quantity in the roots; NaK, Na/K ratio; MDA, malondialdehyde.

In addition to plant growth, salinity affected the physiology of the eight cultivars. In order to identify the main physiological parameters involved in salt tolerance in *A. cruentus* and to differentiate the cultivars, principal component analysis (PCA) and correlation plots were used, as shown in Figure 3 and Figure 4.

Axis 1 and axis 2 of the PCA explained 55.4% of the variance (Figure 3). Axis 1 of the PCA separated plants in control conditions from those exposed to salt (Figure 3a). The sodium contents in leaves, stem and roots and Na/K ratios in leaves and roots were good positive predictors of salt-treated plants, whereas K and Mg content were negatively correlated with Na content (Figure 3b). Axis 2 was mainly explained by growth parameters. In contrast to NaCl treatments, there was no clear discrimination between cultivars.

Sodium accumulation in the plants affected several parameters, as shown on the correlation matrix (Figure 4). The sodium content in all organs (leaves, stems and roots) was negatively correlated with K content in all organs, Mg and Ca content in leaves, biomass of all organs, pigments (chlorophylls and betaxanthins but not betacyanins), photosynthetic activity (net photosynthetic rate, transpiration rate and stomatal conductance) and slightly with polyphenols in leaves. The Na/K ratio in leaves and roots, but not in stems, was also negatively correlated with all these parameters.

The impacted physiological, biochemical and mineral parameters will be further analyzed below.

### 2.4. Mineral Content

**Figure 5:**
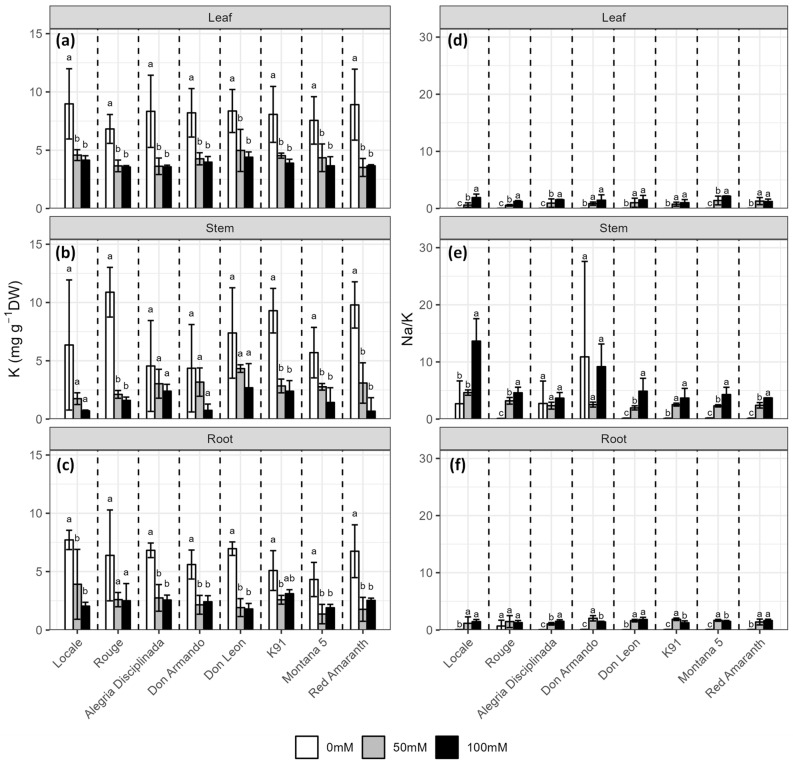
Effect of salinity (0, 50 and 100 mM NaCl) on the potassium content in (**a**) leaves; (**b**) shoot and (**c**) roots and the Na/K ratio in (**d**) leaves; (**e**) shoot and (**f**) roots of the eight *A. cruentus* cultivars. Treatments followed by different letters for the same cultivar are significantly different (*p* < 0.05).

Salt decreased the K content in all organs (*p* < 0.001), but similarly decreased the content at 50 mM and 100 mM NaCl (Figure 5, Table S4). However, this decrease was proportionally higher in stems and roots compared to leaves (Figure 5). In leaves, the highest K reduction was observed in Red Amaranth (−59%) while the lowest was observed in Don Leon (−47%) (Figure 5a). In stems, the reduction in K content ranged from −47% (in Alegria Disciplinada) to −93% (in Red Amaranth), while it ranged from −39% (in K91) to −74% (in Locale and Don Leon) in roots (Figure 5b,c). As a result, salt stress caused an increase in the Na/K ratio in all organs (Figure 5d–f, Table S4). This increase was similar among the cultivars for leaves and roots but depended on the cultivar in stems (Figure 5d–f, Table S4).

**Figure 6:**
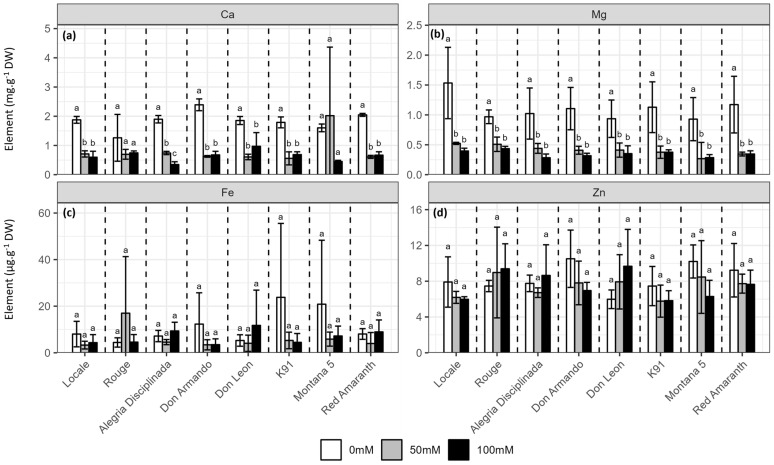
Effect of salinity (0, 50 and 100 mM NaCl) on the (**a**) calcium, (**b**) magnesium, (**c**) iron and (**d**) zinc contents in leaves of the eight *A. cruentus* cultivars. Treatments followed by different letters for the same cultivar are significantly different (*p* < 0.05).

Ca, Fe, Mg and Zn contents were measured in leaves only (Figure 6). A decrease in the Ca content was observed in response to salinity (*p* < 0.001, Figure 6a, Table S4), except in Montana 5. Magnesium content in the leaves of stressed plants decreased by more than 50% compared to the control plants (*p* < 0.001), but again there were no differences between 50 and 100 mM NaCl (Figure 6b, Table S4). Neither Fe (*p* = 0.24) nor Zn (*p* = 0.87) contents were affected by salinity (Figure 6c,d, Table S4).

### 2.5. Photosynthetic Activity in Relation to Sodium Accumulation in Leaves

**Figure 7:**
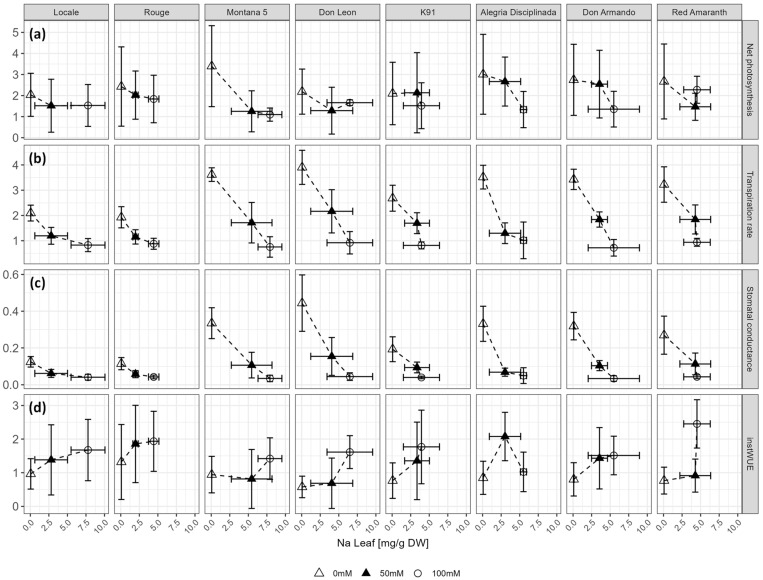
Response of photosynthetic activity to foliar Na accumulation. (**a**) Net photosynthesis (µmol CO_2_ m^−2^ s^−1^); (**b**) transpiration rate (mmol H_2_O m^−2^ s^−1^); (**c**) stomatal conductance (mmol H_2_O m^−2^ s^−1^); (**d**) intrinsic water use efficiency (µmol CO_2_ mmol H_2_O^−1^).

Salt decreased net photosynthesis (A, *p* = 0.0015), stomatal conductance (g_s_, *p* < 0.001) and net transpiration (E, *p* < 0.001), whereas it increased instantaneous water use efficiency (instWUE, *p* < 0.001) (Table S5). Figure 7 shows the photosynthetic parameters in relation to the sodium content in leaves.

The decrease in A was not always linked to Na content in the leaves, depending on the cultivar (Figure 7a). In Montana 5, the decrease in A was proportional to Na accumulation, whereas in most cultivars (Alegria Disciplinada, Don Armando, K91, Rouge), a sharp decrease occurred between 50 mM and 100 mM, despite the modest accumulation of Na in some cultivars (particularly in K91). In Red Amaranth, A was higher at 100 mM compared to 50 mM. In Locale and Don Leon, despite a significant accumulation of Na in the leaves at 100 mM compared to 50 mM NaCl, no decrease in A was observed.

The plant response was cultivar-dependent for E and g_s_ (Figure 7b,c, Table S5). Both parameters strongly correlated (r = 0.976). They decreased proportionally with salt accumulation in Don Armando, Don Leon and Montana 5, whereas they did not differ much between 50 and 100 mM NaCl in Alegria Disciplinada, Locale and Rouge. A sharp decrease in g_s_ and E, despite a modest accumulation of Na between 50 and 100 mM NaCl, occurred in Red Amaranth and K91.

The response of instWUE to salt was less conspicuous compared to A, E and g_s_ (Figure 7). Water use efficiency increased with salt accumulation in most of the cultivars, but only at 100 mM NaCl in Don Leon (Figure 7d). The increase was modest in Locale and Montana 5, whereas it was more marked in Rouge, K91 and Red Amaranth. In Alegria Disciplinada and Don Armando, instWUE increased at 50 mM NaCl but decreased at 100 mM and showed a high standard deviation value.

### 2.6. Biochemical Compound Contents in Relation to Sodium Accumulation in Leaves

**Figure 8:**
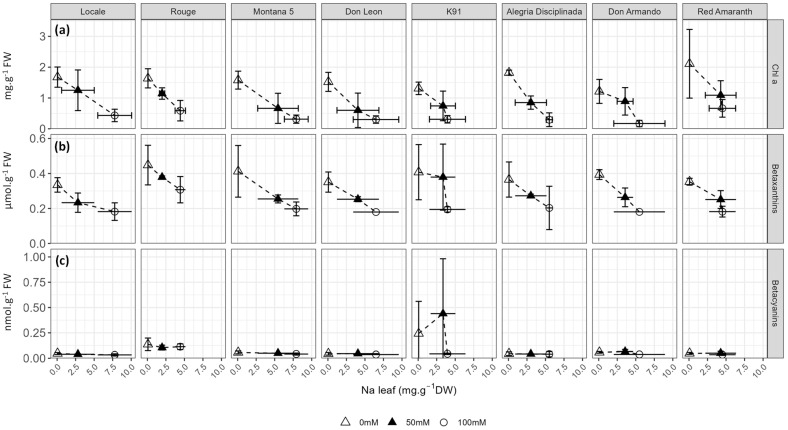
Response of foliar (**a**) chlorophyll *a* (mg·g^−1^ FW), (**b**) betaxanthins (µmol·g^−1^ FW); and (**c**) betacyanins (nmol·g^−1^ FW) to foliar sodium accumulation.

**Figure 9:**
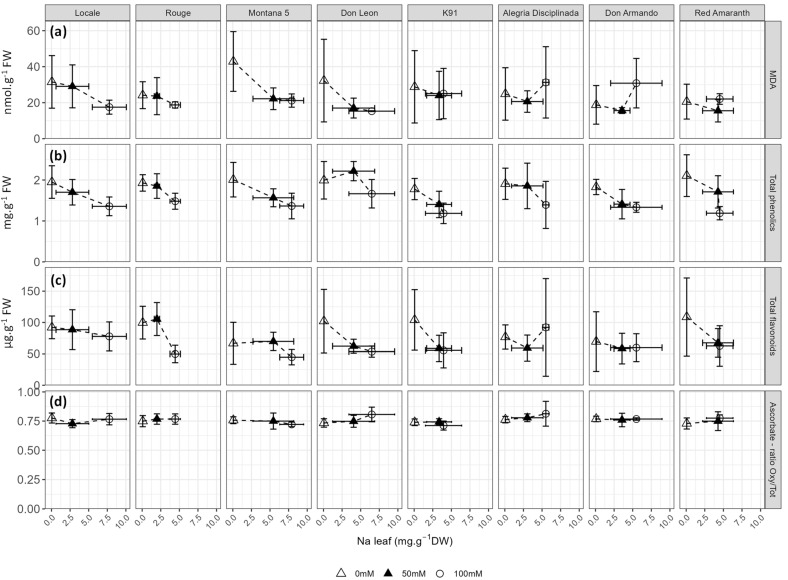
Response of the foliar (**a**) malondialdehyde (MDA, nmol·g^−1^ FW), (**b**) total phenolics (mg·g^−1^ FW), (**c**) total flavonoids (µg·g^−1^ FW) and (**d**) ratio between oxidized and total ascorbate to foliar sodium accumulation.

Pigments (chlorophyll *a*, chlorophyll *b*, betaxanthins and betacyanins) and oxidative stress-related compounds (malondialdehyde (MDA), total flavonoids, total phenolics, ascorbate) were quantified (Figure 8 and Figure 9, Table S6). Betacyanins, MDA and ascorbate were not affected by salt stress, while flavonoids (*p* = 0.018) were only slightly affected (Table S7). In contrast, the concentrations of chlorophyll *a* (*p* < 0.001), chlorophyll *b* (*p* < 0.001), betaxanthins (*p* < 0.001), and total phenolics (*p* < 0.001) strongly decreased with salt stress (Table S7). Since the content of chlorophyll *a* and *b* strongly correlated with one another (r = 0.938), only chlorophyll *a* is presented in Figure 8 and the data of chlorophyll *b* are shown in Table S6.

The content in chlorophylls was strongly reduced by salt in all cultivars at 100 mM NaCl, but only in Alegria Disciplinada, Don Leon and Montana 5 at 50 mM NaCl (Figure 8a, Table S6). Salinity decreased the betaxanthin content by more than 50% in Don Armando, K91 and Montana (Figure 8a). As a result, the chlorophyll and betaxanthin contents in leaves regularly decreased with Na content increase in most cultivars, with the exception of K91, Don Armando and Red Amaranth (Figure 8a,b). In contrast, the concentration of betacyanins was not affected by the Na concentration in the leaves, except in K91 (Figure 8c).

Although it was not affected by salt stress and did not significantly vary among cultivars (Table S7), MDA response to sodium accumulation in the leaves was cultivar-dependent (Figure 9a). The response of total polyphenol and flavonoid content to sodium concentrations in the leaves was similar in most cultivars, with the exception of Don Leon and Alegria Disciplinada (Figure 9b,c). For most cultivars, the concentration of polyphenols decreased proportionally to the Na content in leaves. Some exceptions were nevertheless observed. For example, in Red Amaranth, the foliar Na content was roughly the same at 50 and 100 mM NaCl but a decrease in phenolics was observed (Figure 9b). In Don Armando and Don Leon, the Na concentration in the leaves of plants treated at 50 mM NaCl had no effect or even a positive effect on the phenolic content, respectively (Figure 9b). The ratio between oxidized and total ascorbate was similar whatever the Na content in all cultivars (Figure 9d).

## 3. Discussion

In this study, we compared the tolerance of eight cultivars of *Amaranthus cruentus* to 50 and 100 mM NaCl in hydroponic conditions at the vegetative stage. Our results revealed different levels of tolerance and various physiological responses among the cultivars. Sodium accumulated in all plant organs regardless of the cultivar. Since the salt treatments were applied for four weeks, amaranth plants were subjected to both osmotic and ionic phases of salt stress. Indeed, it was previously observed that both phases of salt stress were detected a couple of days after stress imposition in amaranth [31].

### 3.1. Variability in Salt Tolerance among Leaf and Seed Cultivars of A. cruentus

Our results showed that *A. cruentus* plants were more affected by salt treatments than by the cultivars. This pattern had been observed in other studies screening genotypes for abiotic stresses. In a study of salinity resistance in 25 African rice cultivars (*Oryza glaberrima*), Prodjinoto et al. also found that plants were better discriminated by salt dose than by genotype [63]. Similarly, in a comparison of 12 Tartary buckwheat (*Fagopyrum tataricum*) cultivars, the response to water and heat stress was better explained by the differences between plants than the cultivar [64].

Despite this, differences between cultivars were highlighted in this study. Some cultivars such as Locale, Don Leon and K91 were tolerant to a moderate amount of salinity, given the similar biomass production at 0 and 50 mM NaCl. However, Montana 5, Red Amaranth and Don Armando were nearly as tolerant at 50 mM than at 100 mM NaCl. We also observed that salt tolerance at 50 mM NaCl was not correlated with salt tolerance at 100 mM, meaning that a cultivar tolerant to a moderate salt dose is not always tolerant to a high salt dose in *A. cruentus*. In a previous study that investigated the salt tolerance of several leaf cultivars of *A. cruentus* after 2 weeks of NaCl treatment, Rouge was identified as salt-tolerant, while Locale was identified as more salt-sensitive compared to other *A. cruentus* cultivars [28]. Our results showed that Rouge accumulated less sodium in leaves and stems than Locale after 4 months of stress, which could explain its higher tolerance. However, both produced an equivalent shoot biomass in this experiment, despite being slightly higher in Rouge at 100 mM NaCl. Amaranth species are usually considered to be salt tolerant [28,29,39] and most cultivars tested in this study survived at 100 mM NaCl, demonstrating the value of growing *A. cruentus* in areas moderately affected by salt. The closely related species *Amaranthus hypochondriacus* was tested for drought and multi-salinity (NaCl, CaCl_2_, KCl, MgCl_2_, MgSO_4_) tolerance in field conditions in South Italy [39,40]. It was shown that this grain amaranth can be grown under conditions of moderate combined drought and saline stress, at the cost of a decrease in seed nutritional quality.

Genetic diversity is an important prerequisite for breeding for salt tolerance [65]. Here, we report a noticeable variability in the physiological response (photosynthetic activity and biochemical activity) of *A. cruentus* cultivars to moderate salt stress, paving the way for developing salt-tolerant lines.

### 3.2. Putative Physiological Role of Sodium in Amaranth

Our results showed that the salt response of *A. cruentus* may differ according to the NaCl concentration. A previous study on *A. cruentus* leaf cultivars found that a low concentration of NaCl in hydroponic conditions could stimulate several parameters related to mineral content and oxidative status [34]. After two weeks of exposure, 30 mM NaCl had a positive effect on the plant growth and health compared to control conditions, whereas higher concentrations (60 and 90 mM) had detrimental effects [34]. After 4 weeks of exposure to 50 and 100 mM NaCl, the results of the present experiment differed substantially. Even though some parameters were not affected at 50 mM NaCl in some cultivars, or in some cases slightly up-regulated, generally, no positive effect of salt was observed on any parameter in our study. Previous works on *A. tricolor* demonstrated that similar NaCl concentrations (50–100 mM) had a positive effect on the nutritional quality (several minerals, macronutrients and phenolics) of leaves [41,42]. Often considered as a “functional nutrient” rather than as an essential nutrient in plants (e.g., possible substitution of K in some metabolic functions), Na is required in a small quantity in some NAD-ME-type C_4_ plants for pyruvate transport and conversion of some metabolic intermediates [66,67,68,69]. It was also demonstrated that Na is important in amaranth (*A. tricolor*) besides its putative role in C_4_ photosynthesis, for instance by stimulating N assimilation [70,71,72,73]. Further research using lower salt concentrations could determine the range of NaCl concentrations that stimulates *A. cruentus* growth and the nutritional quality of leaves.

### 3.3. Impact of Sodium Accumulation on Photosynthetic Activity

The eight cultivars investigated in this study could be distinguished based on the accumulation of Na in leaves, especially the accumulation difference between 50 mM and 100 mM NaCl, and its consequence on photosynthetic activity. The salt accumulation in leaves caused by salt stress was linked to an important decrease in stomatal conductance, transpiration rate and, to a lesser extent, carbon assimilation in some cultivars, whereas some others maintained their photosynthetic activity despite foliar sodium accumulation.

The significant water use efficiency increase in stressed plants was caused by a stronger decrease in the transpiration rate compared to the salt-induced decrease in net photosynthesis. Omamt et al. investigated the effect of saline stress on the WUE of various *A. cruentus*, *A. hypochondriacus* and *A. tricolor* genotypes [30]. They observed an increase of about 50% in instantaneous WUE in *A. cruentus* at 100 mM NaCl. However, the values recorded in their study were 2–3 times higher than what we observed in the current study, even in control conditions. The same authors demonstrated that the decrease in photosynthetic activity was, at least in part, due to salt-induced stomatal closure and decrease in stomatal density [30]. An increase in WUE in *A. cruentus* cv. Locale was also observed by Gandonou et al. [29]. An increase in WUE is considered as a salt-tolerance mechanism, improving the capacity of the plant to limit water loss despite the salinity toxic effects [74,75]. Liao et al. reported an increase in WUE in maize in combined water and salt stress conditions [76]. These authors identified salt-induced osmotic adjustment in stomata as the main mechanism of stomatal conductance regulation, resulting in an increased salt tolerance in this C_4_ crop. In our study, the highest WUE under salinity was observed in K91 and Red Amaranth, suggesting that these cultivars were able to maintain photosynthesis and limit transpiration and thus showed a higher salt tolerance from the physiological point of view. In grasses, halophytism has been associated with C_4_ photosynthesis, which could be explained by the high WUE provided by this type of carbon fixation [77]. This could be also true in amaranths, since all species use C_4_ photosynthesis [45].

Another parameter often considered as a reliable physiological index for the tolerance to salt stress is the Na/K ratio, which significantly increased in all organs in our study [78]. In contrast to Na, K is an essential element used in different functions, such as in various steps of cell metabolism, photosynthesis and turgor pressure maintenance [79]. Maintaining a low Na/K ratio in plants and mainly in leaves is thus necessary. Red Amaranth and K91 showed the lowest Na/K ratios explained by their ability to restrict Na accumulation in leaves at 100 mM compared to 50 mM NaCl. Indeed, those cultivars were among the most tolerant at 100 mM NaCl.

### 3.4. Foliar Biochemical Activity Response to Salt Stress

Malondialdehyde is a by-product of lipid peroxidation, caused by reactive oxygen species, which are produced in response to stress, including salt stress [62,80,81]. In our experiment, foliar MDA content did not increase in response to salinity, suggesting low salt-induced oxidative stress on lipids in leaves of the selected *A. cruentus* cultivars. We observed that the total foliar polyphenol content (flavonoids also, to a lesser extent) decreased in plants exposed to NaCl. However, the phenylpropanoid pathway is often upregulated in response to many abiotic stresses because polyphenols have protective roles, for instance against oxidative stress [82,83]. Moreover, no difference in the ratio between oxidized and total ascorbate was observed between the control and stressed plants, although this metabolite is also involved in oxidative stress mitigation [84]. The low intensity of oxidative stress suggested by the low MDA content could explain why the plants exposed to salinity did not upregulate polyphenols and ascorbate production. Betalains, which include the two subfamilies of pigments betaxanthins and betacyanins, are also involved in plant tolerance to abiotic stress. Salinity-induced betalain accumulation has been described in two halophyte species of the Amaranthaceae family [85]. Sarker and Oba (2018) reported an increase in betacyanin and betaxanthin content in the leaves of *A. tricolor* exposed to 200 mM NaCl [86]. A photoprotective role of betacyanins was also described in *A. cruentus* [87]. Here, only the betaxanthin content significantly increased in response to stress.

### 3.5. Differences between Leaf and Seed Cultivars

There are two types of amaranth cultivars, depending on the harvested parts. African Rouge and Locale cultivars have been bred for leaf production, whereas the others are cultivated for their seeds. Even though the cultivar types are clearly distinguishable by several morphological traits (size and color of seeds, shape of leaves, branching pattern or inflorescence structure (see Table S1)), their physiological response to salinity stress did not differ at the vegetative stage. Only the transpiration rate was, on average, 1.5× higher in the grain cultivars than in leaf cultivars in the control conditions and at 50 mM NaCl, although it dropped at the same level at 100 mM NaCl. Comparing leaf and grain cultivars at the reproductive stage could reveal additional differences in response to salt stress. Although amaranths are usually used either for grain production or leaf production, the dual use of leaves and seeds on a single cultivar seems promising on the basis of several defoliation experiments [51,52,53,88,89,90]. To our knowledge, the tolerance of amaranth to defoliation under abiotic stress has not been investigated yet.

### 3.6. Effect of Salt of the Nutritional Quality of Amaranth

Amaranths are recognized for their exceptional nutritional value, both of their leaves and seeds [34,54,56,86,91]. Adverse environmental conditions could alter the nutritional quality of plants, although it was demonstrated that *A. tricolor* keeps its nutritional value under abiotic stress [86,92]. Although the effect of salinity on the nutritional quality was not a main objective of this work, we showed that salt stress caused a decrease in phenolic content in most cultivars. These metabolites, however, have nutritional and health benefits [93]. The decrease in the foliar content of several essential minerals (K, Ca, Mg) should also be highlighted. Given the outstanding nutritional value of this crop, understanding the effects of moderate salt stress on the nutritional quality of *A. cruentus* leaves is crucial and needs further research. A previous work on the two leaf cultivars demonstrated an increase in Mg, P, Fe, vitamin C, phenolic, α-tocopherol and carotenoid contents in leaves when exposed to 30 mM of NaCl, particularly in Rouge [34]. Salt stress and drought stress, the latter being in some respects similar to salt stress, can also improve the nutritional quality of amaranth leaves [42,94]. To our knowledge, the effect of abiotic stress, particularly salinity, on the nutritional quality of the seeds of grain amaranth has not been investigated extensively yet. More broadly, the effect of salt on the reproduction of amaranths requires further research.

## 4. Materials and Methods

### 4.1. Plant Material and Growth Conditions

Leafy cultivars Rouge and Locale were kindly provided by Dr. Christophe B. Gandonou (University of Abomey-Calavi, Cotonou, Benin) and selected based on previous works [28]. Since no information was available about the salt tolerance of grain cultivars, six grain cultivars differing by their origin were randomly selected and obtained from the Genebank of the Crop Research Institute (CRI, Prague, Cezch Republic) (see Table S1 for accession numbers).

Plants were cultivated in greenhouses (SeFy, UCLouvain) at 23–25 °C at day, 20–22 °C at night and 65% RH, under a 16 h photoperiod. When necessary, artificial light was provided by 650 W red-blue LumiGrow LED lights (minimum light intensity of 150 µmol m^−2^ s^−1^). Seeds were sown in 2/3 peat compost (DCM, Amsterdam, The Netherlands) and 1/3 river sand (Mpro, Wavre, Belgium) (volume:volume). Two weeks later, seedlings were transplanted individually in 6 × 6 × 6 cm plastic pots in 2/3 peat compost +1/3 river sand. Two weeks later, they were transplanted in 15 L plastic tanks filled with Hoagland nutritive solution (5 mM KNO_3_, 5.5 mM Ca(NO_3_)_2_, 1 mM NH_4_H_2_PO_4_, 0.5 mM MgSO_4_, 25 µM KCl, 10 µM H_3_BO_4_, 1 µM MnSO_4_, 0.25 µM CuSO_4_, 1 µM ZnSO_4_, 10 µM (NH_4_)_6_Mo_7_O and 1.87 g L^−1^ Fe-EDTA, and pH 5.5–6), with 1 seedling of each cultivar per tank (9 plants/tank). Tanks were randomly assigned to 0, 50 or 100 mM NaCl, with 9 replicates (27 tanks). The NaCl was added in the Hoagland solution and salt stress started 9 days after the transfer to plastic tanks. The nutritive solution was renewed once a week. The experiment took place over 53 days in November and December 2020.

### 4.2. Biomass and Harvest

Plants were harvested 53 days after sowing for destructive measurements, mineral and biochemical analyses. For three plants per cultivar and salt treatment, three young but well-expanded leaves were harvested in liquid nitrogen and stored at −80 °C for further biochemical analyses (see below). For five other plants per cultivar and treatment, the stems, leaves and roots were separated, weighted (for fresh weight), dried at 60 °C for 72 h, then weighed again (for dry weight). Water content was calculated as (fresh weight − dry weight)/fresh weight. Dry material was used for mineral analyses (see below). The salt tolerance index was calculated as the ratio between the mean total biomass production in salt conditions relative to the total biomass production in control conditions [78].

### 4.3. Biochemical Analyses

#### 4.3.1. Pigments

Chlorophyll *a* and *b* were quantified in the leaves of three plants per cultivar and treatment according to [95]. Briefly, 1.2 mL of 80% acetone (*v*/*v*) was added to 50 mg of finely ground (in liquid nitrogen) fresh leaves. After 60 min of incubation at 4 °C, tubes were centrifugated (10,000× *g*, 4 °C, 10 min). A second identical extraction was performed on the pellet; supernatants were combined. Absorbance of the supernatant was read at 663.2 and 646.8 nm (UV-1800 spectrophotomer, Shimadzu, Kyoto, Japan). Chlorophyll *a* and *b* contents were measured as follows: Chl *a* (mg/L) = 12.25 × Abs_663.2_ − 2.79 × Abs_646.8_ and Chl *b* (mg/L) = 21.50 × Abs_646.8_ − 5.10 × Abs_663.2_.

Betalains were extracted in deionized water overnight. Absorbance of the supernatant was read at 540 nm (betacyanins) and 475 nm (betaxanthins) (UV-1800 spectrophotomer, Shimadzu, Kyoto, Japan). Molar extinction coefficients of 62 × 10^6^ cm^2^ mol^−1^ and 48 × 10^6^ cm^2^ mol^−1^, respectively, were used to quantify the pigment content [96,97].

#### 4.3.2. Phenolics

Total phenolics and flavonoids were quantified in the leaves of three plants per cultivar and treatment according to [98,99]. After grounding in liquid nitrogen, 1.4 mL of 80% methanol was added to 100 mg of fresh material, before centrifugation (20,000× *g*, 20 min, 4 °C). The supernatant was stored at −20 °C until quantification.

A volume of supernatant was added to an equal volume of 2% AlCl_3_ for total flavonoid quantification (adapted from [98]). After 10 min of incubation at room temperature in the dark, absorbance was read at 440 nm, with quercetin as the standard.

Total phenolics were quantified as follows: 200 µL of supernatant was added to 2.8 mL of deionized water and 200 µL of Folin–Ciocalteu reagent [99]. Three minutes later, 0.8 mL of 20% Na_2_CO_3_ was added before incubation in a water bath at 40 °C for 40 min. Absorbance was read at 760 nm with gallic acid as the standard.

#### 4.3.3. Malondialdehyde

Malondialdehyde, a marker of lipid peroxidation [100], was quantified in the leaves of three plants per cultivar and treatment according to [101]. It was extracted in 250 mg of finely ground fresh leaves with 4 mL of 5% trichloroacetic acid with 1.25% glycerol. After 5 min of incubation at 4 °C, tubes were centrifuged for 10 min at 4 °C, 12,000× *g*. Then, 2 mL of supernatant was added to 2 mL of a 0.67% aqueous solution of thiobarbituric acid. Samples were incubated for 30 min in a water bath at 100 °C. Absorbance was read at 532 and 600 nm. Malondialdehyde concentration (mM) was calculated as (A_532nm_ − A_600_)/155 mM cm^−1^ [101].

#### 4.3.4. Ascorbate

Ascorbate was quantified with some adaptations from [102], in the leaves of three plants per cultivar and treatment. Briefly, it was extracted in 250 mg of finely ground fresh leaves with 4 mL of 5% trichloroacetic acid (TCA). After 15 min of incubation on ice, samples were centrifugated (5 min, 4 °C, 10,000× *g*). Next, 200 µL of supernatant was added to 400 µL of phosphate buffer (0.2 M, pH 7.4). For oxidized ascorbate quantification, 0.4 mL of water was added. For total ascorbate quantification, 200 µL of 10 mM 2,2′-dithiothreitol was added, the samples were incubated 5 min at room temperature, then 200 µL of 0.5% N-ethylmaleimide was added. After 1 min of incubation at room temperature, 1 mL 10% TCA was added in all the tubes, then 0.8 mL 42.5% H_3_PO_4_ and 0.8 mL 4% dipyridyl were added. Finally, 400 µL of 3% FeCl_3_ was added while agitating. Tubes were incubated 60 min in a water bath at 37 °C. Absorbance was read at 525 nm. Ascorbic acid was used as the standard.

#### 4.3.5. Mineral Content

The concentrations of Na and K were quantified in the roots, stem and leaves of three plants per cultivar and treatment while the concentrations of Ca, Fe, Mg and Zn were quantified only in the leaves. For mineralization, 4 mL of 68% nitric acid was added to 50–100 mg of ground dry plant material. After one night of incubation, nitric acid was evaporated using a sand bath. Then, 1.5 mL of aqua regia (500 µL of 68% nitric acid and 1.5 mL of 37% hydrochloric acid) was added and incubated two minutes on the sand bath. The volume was adjusted to 10 mL with deionized water before filtration on Whatman Grade 1 paper. The concentration of Na, K, Mg, Ca, Fe and Zn was determined by atomic absorption spectroscopy (ICE 3300, Thermo Scientific, Waltham, MA, USA) after the required dilutions and addition of 1% LaCl_3_ for Na and K quantification.

### 4.4. Photosynthetic Activity

The portable photosynthesis system LCpro-SD (ADC Bioscientific Ltd., Hoddesdon, United Kingdom) was used for photosynthetic activity analyses. Measurements were performed on three plants per cultivar and treatment, 51 days after sowing (27 days after stress). Net photosynthesis (A), stomatal conductance (g_s_) and transpiration rate (E) were recorded in a young, well-expanded leaf after several minutes of stabilization, in conditions of ambient irradiance, carbon dioxide concentration, air humidity and temperature, similarly to [103]. Water use efficiency was calculated as A/E.

### 4.5. Statistical Analyses

All statistical analyses were performed in R, version 4.2.1 [104]. Normality of the data was verified based on histograms of residuals and homoscedasticity was verified with a Levene test. For each variable (plant growth, mineral content, physiological and biochemical parameters), analyses of variance (ANOVA 2) were performed with the salt treatment and cultivars as the fixed factors using the function “aov()” (base R). Detailed results of ANOVA 2 are presented in Tables S2, S4, S5 and S7. For each variable, Tukey’s test from base R was used to perform multiple comparison tests to evaluate the differences between salt treatments within each cultivar. The package *ade4* was used to perform a principal component analysis to visualize the differences among the cultivars and salt treatments according to mineral and biomass data. Redundant variables with r > 0.8 were removed prior to analysis. Correlations between all the variables were quantified with the Pearson correlation coefficient (r) and significance tests were used to calculate the associated *p*-values with R package *corrplot*. Data are presented in figures and tables as the mean ± standard deviation, with adequate rounding.

## 5. Conclusions

Our results demonstrate a noticeable tolerance of *A. cruentus* cv. Don Leon and K91 at the vegetative stage under moderate salt stress (50 mM) in hydroponic conditions, since biomass production did not differ from the control conditions. Sodium accumulated in all organs regardless of the cultivar, mainly in stems. Different physiological responses to foliar sodium accumulation were observed among the cultivars, suggesting a predominance of the ionic phase when physiology was negatively affected in response to ion accumulation or of the osmotic phase when adverse effects on physiology were observed, despite no or a low sodium accumulation. Water use efficiency increased in response to salt because of an efficient decrease in stomatal conductance, despite a decrease in net photosynthesis. The lower transpiration rate of the leaf cultivars compared to the grain cultivars was the unique discriminating physiological trait between the two types of cultivars. Since salt stress did not increase neither MDA content nor the metabolites involved in protection against radical oxygen species such as polyphenols, betacyanins and ascorbate, we suggest that the oxidative stress was limited. This work provides a basis for further investigation of the physiological mechanisms underlying the variations in salt tolerance in red amaranth, a plant with a promising future in resilient agro-ecosystems under global change.

## Figures and Tables

**Table 1:** Salt tolerance index (STI) of the eight *A. cruentus* cultivars at 50 and 100 mM NaCl.

Cultivar	STI_50mM_	STI_100mM_
Locale	0.759	0.327
Rouge	0.639	0.476
Montana 5	0.595	0.546
Don Leon	0.913	0.521
K91	0.841	0.569
Alegria Disciplinada	0.717	0.264
Don Armando	0.681	0.580
Red Amaranth	0.631	0.617

## Data Availability

Data are contained within the article or Supplementary Material.

## Supplementary Materials

The following supporting information can be downloaded at: https://www.mdpi.com/article/10.3390/plants12183310/s1, Table S1: Origin and main morphological characteristics of the eight *A. cruentus* cultivars used in this study; Table S2: Anova results of biomass production; Table S3: Water content of leaves, stem and roots of eight cultivars of *A. cruentus* exposed to 0 mM, 50 mM and 100 mM of NaCl; Table S4: Anova results of mineral content; Table S5: Anova results of photosynthetic activity; Table S6: Foliar content of chlorophyll *b* of eight cultivars of *A. cruentus* exposed to 0 mM, 50 mM and 100 mM of NaCl; Table S7: Anova results of biochemical data.
