# Supplementary material for: Comparison of Salt Stress Tolerance among Two Leaf and Six Grain Cultivars of Amaranthus cruentus L
Source: Plants (Basel). 2023 Sep 19;12(18):3310. doi: 10.3390/plants12183310 (PMC10535409; doi:10.3390/plants12183310)
Supplement: Supplementary file 1 [file plants-12-03310-s001.zip › plants-2542340-supplementary.pdf]

Table S1. Origin and main morphological characteristics of the eight *A. cruentus* cultivars used in this study.

Cultivar type	Cultivar name	Country	Accession*	Red pigmentation	Leafy ramifications	Inflorescence color	Seed color	Starch type	1000 seeds weight (mg)
Leafy	Locale	Benin	/	No	Yes	Green-Yellow	Black	Non-waxy	390 ± 6.36
Leafy	Rouge	Benin	/	Yes	Yes	Red	Black	Non-waxy	331 ± 26.9
Grain	Alegria Disciplinada	Mexico	01Z5200138	Yes	No	ND*	White	Waxy	761 ± 123
Grain	Don Armando	USA	01Z5200035	Yes	No	Reddish yellow	White	Waxy	749 ± 36.5
Grain	Don Leon	Argentina	01Z5200166	No	No	Green-Yellow	White	Waxy	802 ± 91.8
Grain	K91	Venezuela	01Z5200094	Yes	Yes	Red	White	Non-waxy	608 ± 24.6
Grain	Montana 5	USA	01Z5200032	Yes	No	Green and red	White	Waxy	521 ± 164
Grain	Red Amaranth	China	01Z5200162	Yes	No	Reddish yellow	White	Waxy	645 ± 92.9

* in the Genebank of the Crop Research Institute (CRI, Prague, Cezch Republic). ND : no data.

Table S2. Anova results of biomass production.

variable	term	df	sumsq	meansq	statistic	p.value	signif
Leaf dry weight	var	7	2,4	0,3	2,5482	0,0189	*
	trt	2	20,5	10,3	75,9123	< 0,001	***
	var:trt	14	4,1	0,3	2,1492	0,0154	*
	Residuals	96	13,0	0,1			
Leaf fresh weight	var	7	147,0	21,0	2,6072	0,0166	*
	trt	2	1858,9	929,5	115,3931	< 0,001	***
	var:trt	14	217,2	15,5	1,9259	0,0327	*
	Residuals	96	773,3	8,1			
Leaves water content	var	7	0,0	0,0	1,0713	0,3879	ns
	trt	2	0,1	0,1	9,3639	0,0002	***
	var:trt	14	0,1	0,0	0,8746	0,5880	ns
	Residuals	96	0,6	0,0			
Root dry weight	var	7	0,4	0,1	3,1709	0,0046	***
	trt	2	0,7	0,4	20,9021	< 0,001	***
	var:trt	14	0,2	0,0	0,7727	0,6956	ns
	Residuals	96	1,6	0,0			
Root fresh weight	var	7	223,5	31,9	5,9796	< 0,001	***
	trt	2	617,4	308,7	57,8058	< 0,001	***
	var:trt	14	65,9	4,7	0,8817	0,5805	ns
	Residuals	96	512,7	5,3			
Root water content	var	7	0,0	0,0	1,0395	0,4089	ns
	trt	2	0,0	0,0	3,1061	0,0493	*
	var:trt	14	0,0	0,0	1,3725	0,1816	ns
	Residuals	96	0,1	0,0			
Stem dry weight	var	7	2,0	0,3	6,4061	< 0,001	***
	trt	2	2,2	1,1	24,5931	< 0,001	***
	var:trt	14	0,8	0,1	1,2128	0,2792	ns
	Residuals	95	4,2	0,0			
Stem fresh weight	var	7	321,6	45,9	8,7797	< 0,001	***
	trt	2	959,0	479,5	91,6353	< 0,001	***
	var:trt	14	72,6	5,2	0,9910	0,4685	ns
	Residuals	96	502,4	5,2			
Stem water content	var	7	0,0	0,0	1,9093	0,0764	ns
	trt	2	0,0	0,0	5,8681	0,0040	***
	var:trt	14	0,0	0,0	1,2906	0,2277	ns
	Residuals	95	0,0	0,0			
Total dry weight	var	7	9,7	1,4	3,3988	0,0028	***
	trt	2	45,4	22,7	55,6513	< 0,001	***
	var:trt	14	9,9	0,7	1,7387	0,0604	ns
	Residuals	95	38,8	0,4			

Table S3. Water content of leaves, stem and roots of eight cultivars of *A. cruentus* exposed to 0 mM, 50 mM and 100 mM of NaCl. Treatments followed by different letters for the same cultivar are significantly different (α = 0.05).

Cultivar	0 mM	50 mM	100 mM	0 mM	50 mM	100 mM
	Leaves water content			Stem water content		
Locale	0.89 ± 0.01 a	0.87 ± 0.01 a	0.83 ± 0.03 b	0.95 ± 0.00 a	0.95 ± 0.01 a	0.95 ± 0.01 a
Rouge	0.88 ± 0.01 a	0.86 ± 0.03 ab	0.84 ± 0.00 b	0.95 ± 0.01 a	0.95 ± 0.01 a	0.94 ± 0.01 a
Montana 5	0.88 ± 0.02 a	0.88 ± 0.04 a	0.77 ± 0.22 a	0.94 ± 0.01 a	0.95 ± 0.01 a	0.94 ± 0.01 a
Don Leon	0.89 ± 0.02 a	0.88 ± 0.01 a	0.73 ± 0.29 a	0.95 ± 0.02 a	0.95 ± 0.01 a	0.94 ± 0.02 a
K91	0.89 ± 0.00 a	0.89 ± 0.02 a	0.87 ± 0.02 a	0.96 ± 0.01 a	0.96 ± 0.01 ab	0.95 ± 0.00 b
Alegria Disc.	0.87 ± 0.02 a	0.88 ± 0.01 a	0.73 ± 0.13 b	0.93 ± 0.02 a	0.94 ± 0.01 a	0.95 ± 0.02 a
Don Armando	0.88 ± 0.01 a	0.89 ± 0.01 a	0.86 ± 0.02 b	0.95 ± 0.0065 ab	0.95 ± 0.01 a	0.93 ± 0.01 b
	Roots water content					
Locale	0.97 ± 0.01 a	0.97 ± 0.02 a	1.00 ± 0.05 a			
Rouge	0.967 ± 0.01 b	0.97 ± 0.01 b	1.00 ± 0.03 a			
Montana 5	0.97 ± 0.02 a	0.98 ± 0.02 a	0.97 ± 0.01 a			
Don Leon	0.97 ± 0.01 a	0.97 ± 0.01 a	1.03 ± 0.11 a			
K91	0.97 ± 0.01 a	0.97 ± 0.01 a	0.98 ± 0.02 a			
Alegria Disc.	0.97 ± 0.01 a	0.97 ± 0.01 a	0.97 ± 0.02 a			
Don Armando	0.97 ± 0.02 a	0.97 ± 0.01 a	0.95 ± 0.02 a			
Red Amaranth	0.97 ± 0.01 a	0.98 ± 0.01 a	0.96 ± 0.02 a			

Table S4. Anova results of mineral content.

		term	df	sumsq	meansq	statistic	p.value	signif
Leaf	Ca	var	7	1,1	0,2	0,32	0,9455	ns
		trt	2	11,5	5,8	11,91	< 0,001	***
		var:trt	14	4,9	0,4	0,73	0,7425	ns
		Residuals	120	58,0	0,5			
	Fe	var	7	202,8	29,0	0,40	0,9034	ns
		trt	2	212,6	106,3	1,45	0,2383	ns
		var:trt	14	1079,5	77,1	1,05	0,4072	ns
		Residuals	120	8787,8	73,2			
	K	var	7	8,3	1,2	0,14	0,9950	ns
		trt	2	157,1	78,6	9,22	< 0,001	***
		var:trt	14	5,7	0,4	0,05	1,0000	ns
		Residuals	120	1022,8	8,5			
	Mg	var	7	0,3	0,0	0,32	0,9442	ns
		trt	2	4,9	2,5	17,26	< 0,001	***
		var:trt	14	0,3	0,0	0,15	0,9998	ns
		Residuals	118	16,9	0,1			
	Na	var	7	25,0	3,6	0,77	0,6152	ns
		trt	2	278,8	139,4	29,97	< 0,001	***
		var:trt	14	31,5	2,2	0,48	0,9379	ns
		Residuals	118	548,8	4,7			
	NaK	var	7	3,8	0,5	2,65	0,0141	*
		trt	2	54,2	27,1	132,05	< 0,001	***
		var:trt	14	5,4	0,4	1,87	0,0359	*
		Residuals	118	24,2	0,2			
	Zn	var	7	32,9	4,7	0,29	0,9578	ns
		trt	2	4,7	2,3	0,14	0,8669	ns
		var:trt	14	70,8	5,1	0,31	0,9919	ns
		Residuals	120	1960,4	16,3			
Stem	K	var	7	29,8	4,3	0,53	0,8096	ns
		trt	2	246,1	123,0	15,33	< 0,001	***
		var:trt	14	66,4	4,7	0,59	0,8674	ns
		Residuals	119	955,0	8,0			
	Na	var	7	18,7	2,7	0,23	0,9764	ns
		trt	2	354,5	177,3	15,50	< 0,001	***
		var:trt	14	26,8	1,9	0,17	0,9997	ns
		Residuals	117	1338,0	11,4			
	NaK	var	7	667,0	95,3	6,08	< 0,001	***
		trt	2	386,8	193,4	12,35	< 0,001	***
		var:trt	14	501,6	35,8	2,29	< 0,001	***
		Residuals	113	1770,1	15,7			
Root	K	var	7	11,1	1,6	0,28	0,9596	ns
		trt	2	125,3	62,6	11,15	< 0,001	***

		var:trt	14	12,2	0,9	0,16	0,9998	ns
		Residuals	120	674,0	5,6			
	Na	var	7	5,9	0,8	0,30	0,9519	ns
		trt	2	63,2	31,6	11,40	< 0,001	***
		var:trt	14	10,3	0,7	0,27	0,9963	ns
		Residuals	120	332,7	2,8			
	NaK	var	7	1,8	0,3	1,31	0,2543	ns
		trt	2	59,3	29,6	147,35	< 0,001	***
		var:trt	14	6,1	0,4	2,15	0,0138	*
		Residuals	113	22,7	0,2			

Table S5. Anova results of photosynthetic activity.

variable	term	df	sumsq	meansq	statistic	p.value	signif
Net photosynthesis	var	7	8,825	1,261	0,6445	0,7181	ns
	trt	2	27,200	13,600	6,9518	0,0015	***
	var:trt	14	15,067	1,076	0,5501	0,8964	ns
	Residuals	96	187,805	1,956			
Stomatal conductance	var	7	0,233	0,033	9,1310	< 0,001	***
	trt	2	1,104	0,552	151,1021	< 0,001	***
	var:trt	14	0,262	0,019	5,1243	< 0,001	***
	Residuals	96	0,351	0,004			
Transpiration rate	var	7	12,559	1,794	7,9510	< 0,001	***
	trt	2	99,113	49,556	219,6132	< 0,001	***
	var:trt	14	11,088	0,792	3,5098	< 0,001	***
	Residuals	96	21,663	0,226			
instWUE	var	7	5.587	0.798	1.353	0.2353	ns
	trt	2	12.651	6.326	10.73	< 0,001	***
	var:trt	14	10.21	0.729	1.237	0.2643	ns
	Residuals	88	51.90	0.590			

Table S6. Foliar content of chlorophyll *b* of eight cultivars of *A. cruentus* exposed to 0 mM, 50 mM and 100 mM of NaCl. Treatments followed by different letters for the same cultivar are significantly different ($\alpha = 0.05$).

Cultivar	0 mM	50 mM	100 mM
Locale	0.416 ± 0.077 a	0.304 ± 0.181 ab	0.109 ± 0.062 b
Rouge	0.408 ± 0.076 a	0.275 ± 0.042 b	0.134 ± 0.071 c
Montana 5	0.385 ± 0.044 a	0.163 ± 0.126 b	0.092 ± 0.067 b
Don Leon	0.386 ± 0.075 a	0.151 ± 0.136 b	0.078 ± 0.030 b
K91	0.453 ± 0.241 a	0.181 ± 0.116 ab	0.085 ± 0.029 b
Alegria Disc.	0.447 ± 0.037 a	0.189 ± 0.059 b	0.081 ± 0.032 c
Don Armando	0.314 ± 0.103 a	0.217 ± 0.108 ab	0.067 ± 0.011 b
Red Amaranth	0.479 ± 0.239 a	0.253 ± 0.113 ab	0.155 ± 0.066 b

Table S7. Anova results of biochemical data.

variable	term	df	sumsq	meansq	statistic	p.value	signif
Ascorbate - ratio Oxy/Tot	var	7	0,0	0,0	1,0294	0,4243	ns
	trt	2	0,0	0,0	0,6090	0,5483	ns
	var:trt	14	0,0	0,0	0,6676	0,7924	ns
	Residuals	45	0,1	0,0			
Betacyanins	var	7	0,2	0,0	2,0139	0,0820	ns
	trt	2	0,0	0,0	1,2468	0,3002	ns
	var:trt	14	0,2	0,0	0,7537	0,7078	ns
	Residuals	34	0,5	0,0			
Betaxanthins	var	7	0,1	0,0	1,4543	0,2167	ns
	trt	2	0,3	0,2	25,2233	< 0,001	***
	var:trt	14	0,0	0,0	0,2654	0,9947	ns
	Residuals	34	0,2	0,0			
Chl a	var	7	2,4	0,3	2,1088	0,0605	ns
	trt	2	18,1	9,1	56,1033	< 0,001	***
	var:trt	14	1,0	0,1	0,4536	0,9462	ns
	Residuals	48	7,8	0,2			
Chl b	var	7	0,1	0,0	1,0594	0,4038	ns
	trt	2	1,2	0,6	42,2679	< 0,001	***
	var:trt	14	0,1	0,0	0,4527	0,9467	ns
	Residuals	48	0,7	0,0			
chl_ratio	var	7	7,5	1,1	0,8454	0,5559	ns
	trt	2	0,7	0,4	0,2853	0,7531	ns
	var:trt	14	13,5	1,0	0,7598	0,7046	ns
	Residuals	47	59,9	1,3			
MDA	var	7	619,9	88,6	0,6467	0,7152	ns
	trt	2	640,6	320,3	2,3387	0,1073	ns
	var:trt	14	1842,7	131,6	0,9611	0,5049	ns
	Residuals	48	6573,6	137,0			
Total flavonoids	var	7	6155,8	879,4	0,7911	0,5982	ns
	trt	2	9664,1	4832,1	4,3467	0,0183	*
	var:trt	14	11978,3	855,6	0,7697	0,6952	ns
	Residuals	49	54471,2	1111,7			
Total phenolics	var	7	1,5	0,2	1,7831	0,1120	ns
	trt	2	3,9	2,0	16,7340	< 0,001	***
	var:trt	14	0,8	0,1	0,4873	0,9291	ns
	Residuals	49	5,7	0,1			
